# Inhibition Effect of Extract of *Psychotria viridiflora* Stem on α-Amylase and α-Glucosidase and Its Application in Lowering the Digestibility of Noodles

**DOI:** 10.3389/fnut.2021.701114

**Published:** 2021-08-12

**Authors:** Qimin Chen, Joanne Yi Hui Toy, Cynthia Seta, Tiong Chia Yeo, Dejian Huang

**Affiliations:** ^1^Department of Food Science and Technology, National University of Singapore, Singapore, Singapore; ^2^Sarawak Biodiversity Centre, Kuching, Malaysia; ^3^National University of Singapore (Suzhou) Research Institute, Suzhou Industrial Park, Suzhou, China

**Keywords:** *Psychotria viridiflora*, characterisation, proanthocyanidins, noodles, *in vitro* study

## Abstract

A collection of tropical medicinal plants from East Malaysia's rainforests are used by indigenous tribes for their curative properties. Despite their purported healing properties, these forest plant species are largely unexplored and hence remain virtually unknown to the outside world. In this study, antidiabetic properties of *Psychotria viridiflora*, a plant used to treat diabetes by a local community in Sarawak, Malaysia were investigated. Ethyl acetate (EA) extract of *P. viridiflora* stem was found to exhibit high starch hydrolase inhibition activity with an IC_50_ value of 15.4 ± 2.1 μg/ml against porcine α-amylase and an IC_50_ value of 32.4 ± 3.7 μg/ml against rat intestinal α-glucosidase. A complex mixture of A-type oligomeric proanthocyanidins containing (epi)fisetinidol, (epi)afzelechin, (epi)guibourtinidol, and (epi)catechin were found. These compounds may be responsible for the starch hydrolase inhibition activity. Ethyl acetate (EA) extract of *P. viridiflora* stem was incorporated into wheat and rice flour to reformulate noodles with slow digestibility and was assessed under *in vitro* simulated gastrointestinal conditions. A dose-dependent effect on digestibility was observed for both noodles upon incorporation of 1-6% (w/w) of EA extract, with noodles containing 6% (w/w) extract exhibiting the greatest reduction in digestibility. As compared to rice noodles containing 6% extract (31.16% inhibition), wheat noodles with the same extract concentration had a smaller decline in digestibility (27.25% inhibition) after 180 min. Overall, our findings highlight the potential of *P. viridiflora* in the prevention of postprandial hyperglycaemia.

## Introduction

The incidence of type 2 diabetes is increasing at an unprecedented rate worldwide partially due to the increased prevalence of obesity and ageing. The number of type 2 diabetes cases is projected to increase from 415 to 642 million people by 2040 (1). Type 2 diabetes is caused by insulin resistance and an insufficient compensatory insulin secretory response. As a result, starch is rapidly degraded by starch hydrolases after a meal, thereby causing an elevated blood glucose level which is defined as postprandial hyperglycaemia (PPHG) (2). This metabolic disorder is a great concern since a high blood sugar level may lead to complications such as cardiovascular disease, retinopathy, and amputation (3). Currently, antidiabetic drugs such as acarbose are commonly used to prevent postprandial hyperglycemia by slowing down starch digestion. However, the consumption of acarbose may lead to severe side effects such as flatulence, diarrhoea and bloating. This occurrence is due to the accumulation of undigested oligosaccharides, trisaccharides, and disaccharides in the large intestine, which will undergo fermentation by faecal flora to produce hydrogen and methane gas (4). In view of this, there is a need to explore other starch hydrolase inhibitors, most preferably from natural origins that lack the undesired symptoms.

An immense range of botanicals could be found in Southeast Asia and some of these edible and medical plants are used to treat certain diseases since ancient times (5, 6). Local tribes living in remote rainforests in Sarawak, Malaysia rely heavily on a wide assortment of tropical plants for the restoration of health and disease treatments (such as wounds, snakebites, and diabetes) (7). However, few studies have been conducted scientifically to prove the efficacy and safety of these traditional remedies. On a mission to decode the biodiversity in Sarawak rainforest, we (Sarawak Biodiversity Center) have been collecting samples to establish a plant extract library that contains both local plant species and microbial extracts for collaborators all around the world to uncover the chemical and scientific principles behind their purported medical and health-promoting benefits (8). As such, we have developed high throughput assays to study the starch hydrolase inhibitory potentials of the plant extracts (9). Through this screening process, new bioactive constituents with the potential to combat postprandial hyperglycemia will be discovered and subsequently used for the development of low glycemic index starch-rich food. During the initial stage of our collaboration, we conducted a screening of 25 different plant extracts from Sarawak Biodiversity Center for their starch hydrolase inhibition activity and we found that the ethyl acetate extracts of *P. viridiflora* extracts exhibited the strongest α-amylase and α-glucosidase inhibition effects. As such, we further characterised the active constituents of the EA extracts and evaluated their effects as an ingredient in wheat and rice noodles, based on their *in vitro* digestibility. Reported here are our findings.

## Materials and Methods

### Materials

The *P. viridiflora* samples (Voucher specimen: SABC 10761) were collected from the central region of Borneo, Sarawak, Malaysia, 2017. Porcine pancreatic α-amylase (A3176, type VI-B), rat intestinal α-glucosidase (I1630), pancreas lipase type II (L3126), corn starch (S4126), acarbose (A8980), 3,5-dinitrosalicylic acid (DNSA), bile, pancreatin from porcine pancreas (Cat. No. P754526G), pepsin with an activity of ≥250 U/mg from porcine gastric mucosa (Cat. No. P7000-100) were all purchased from Sigma-Aldrich (Saint Louis, Missouri, USA). Amyloglucosidase (3,300 U/ml), α-amylase (thermo-stable, 3,000 U/ml), protease (50 mg/ml, 350 tyrosine U/ml) were purchased from Megazyme. Phosphate buffered Saline and Tris buffer (ultra-pure grade) (PBS) were purchased from Vivantis technologies (Subang Jaya, Selangor, Malaysia). Calcium chloride was purchased from Thermo Fisher Scientific Inc. (Waltham, MA, USA). Plain wheat flour produced in Brussel and white rice flour produced in Thailand were purchased from NTUC Fairprice in Singapore. HPLC grade acetone, acetonitrile and methanol were obtained from Tedia Company (Fairfield, OH, USA). The 96-well polystyrene microplates with flat bottom were purchased from Fisher Scientific (Nunc, Rochester, NY, USA). Microplate reader was purchased from Biotek Instruments Inc. (Winooski, VT, USA). Electrospray ionisation mass spectra (ESI-MS) were obtained from a Finnigan/MAT LCQ ion trap mass spectrometer (San Jose, CA).

### Extraction of the Starch Hydrolase Inhibitors From *P. viridiflora*

Plant extracts were prepared at SBC. The stem of *P. viridiflora* was dried in an oven at 45°C and then ground into a powder. A total of 6.2 kg powder was obtained and sequentially extracted with hexane, dichloromethane, ethyl acetate, methanol, and water. The five extracts were then filtered through Whatman filter paper No-1 and concentrated *via* rotary evaporation and further dried in a vacuum oven to give a dried powder that was stored in the sample bank and for further research.

### Assay of α-Amylase or α-Glucosidase Inhibition Activity

The inhibition activity of the various samples was evaluated using a high throughput method developed in our lab (9). Corn starch (0.5 g) was first dissolved in 25 ml PBS buffer by heating the mixture for 2.5 min at 300 rpm. The corn starch mixture was then cooled to room temperature with continuous stirring at 300 rpm. The α-glucosidase stock solution was first prepared by dissolving 0.5 g of rat intestinal α-glucosidase powder in 20 ml of PBS with constant stirring on an ice-bath for 30 min before undergoing centrifugation at 2,000 g at 4°C for 10 min. On the other hand, a stock solution of α-amylase was prepared by dissolving 4 mg of α-amylase into 1 ml of PBS and subsequently sonicated. During the analysis, 200 μl of α-amylase or α-glucosidase stock solution was diluted in 7.8 ml PBS buffer to give a working solution. In each well, 10 μl of α-amylase or α-glucosidase was mixed with a series concentration of plant extracts (20 μl) or acarbose and subsequently incubated at 37°C for 15 min with medium shaking. After incubation, 60 μl of the corn starch solution was added and the readings were recorded at 660 nm for 1.5 h with one reading per 30 s. The percentage inhibition was determined by Equation 1 where AUC represents the area under the curve. The IC_50_ is the inhibitor concentration required to produce 50% inhibition.

(1) $$\begin{array}{c} \text{Inhibition }(\%)=\frac{\text{AUC inhibitor}-\text{AUC control}}{\text{AUC inhibitor}}\times 100 \end{array}$$

Additionally, the IC_50_ values of the samples were converted into acarbose equivalent (AE) which is defined in equation 2.

(2) $$\begin{array}{c} \text{AE of a sample}=\frac{{\text{IC}}_{50\;}\text{of acarbose}}{{\text{IC}}_{50\;}\text{of a sample}} \end{array}$$

### Characterisation of Compounds by High Performance Liquid Chromatography-Mass Spectrometry

EA extract was subjected to LC-MS analysis using the Bruker Amazon ion trap mass spectrometer equipped with a Dionex ultimate 3000 RS Diode array detector (Billerica, MA, USA). The heated capillary and spray voltage were maintained at 250°C and 4.5 kV, respectively. Additionally, nitrogen was operated at 80 psi for sheath gas flow rate and at 20 psi for the auxiliary gas flow rate. The MS^3^ collision gas used was helium with a collision energy of 30% of the 5 V end-cap maximum tickling voltage. The mass spectra were scanned from *m/z* 100-2,000 in both the positive and negative ion modes with a scan speed at one scan per second. Each fraction was dissolved in methanol at 1 mg/ml and filtered through a 0.45 μl RC membrane. During the HPLC run, 1 μl of fraction was injected into the Develosil diol column (4.6 x 250 mm, Batch: 250612). The mobile phase consists of solvent A, 2% acetic acid in acetonitrile and solvent B, acidic aqueous methanol (CH_3_OH: H_2_O: HOAc, 95:3:2 v/v/v). Elution programme started with 7% B for 3 min, ramping up to 37.6% at 60 min, 100% at 63 min and holding for 7 min at a flow rate at 1.0 ml/min at 25°C.

### Preparation of Rice and Wheat Noodles Incorporated With EA Extracts of *P. viridiflora*

Rice flour (5 g) was incorporated with powdered EA fraction of *P. viridiflora* at different concentrations (0, 1, 3, and 6%) and mixed together with 2% (w/w) salt and 7.5 ml of deionised water. Rice slurries were then spread evenly on a stainless steel tray and steamed in a steamer for 5 min to form cooked noodle sheets of 2 mm thickness. Wheat flour was processed in the same manner but with the addition of only 3.3 ml of deionised water. Wheat dough sheets were subsequently boiled for 5 min. Noodle sheets were then left to cool at room temperature for 15 min. Thereafter, the noodle sheets were cut into noodle strands (2 mm in thickness and 10 mm in width). The moisture content of these noodles was measured according to the AOAC method 934.01 (10).

### *In vitro* Digestion of Noodles

*In vitro* digestion process was based on the procedure introduced earlier (11). First, the noodles were blended and weighed based on their carbohydrate content. Simulated salivary fluid (SSF) was prepared freshly before use according to (12) with the following concentration of salts: 15.1 mM KCl, 3.7 mM KH_2_PO_4_, 13.6 mM NaHCO_3_, 0.15 mM MgCl_2_(H_2_O)_6_, 0.06 mM (NH_4_)_2_CO_3_, 0.75 mM CaCl_2_(H_2_O). The pH was adjusted to 7.0 with 6M HCl. During the oral digestion, 75 U/ml of α-amylase in 1 ml of SSF was added to 0.75 g of the noodle mixture containing 0% of the extract. To stimulate oral digestion, each mixture was vortex for 20 s and placed in a water bath at 37°C for 1 min and 40 s.

Following oral phase digestion, gastric phase digestion was simulated. First, the pH of the mixture was adjusted to 3.0 using 1.0 M HCl. Simulated gastric fluid (SGF) was prepared freshly before use according to (12) with the following concentration of salts: 6.9 mM KCl, 0.9 mM KH_2_PO_4_, 25 mM NaHCO_3_, 47.2 mM NaCl, 0.1 mM MgCl_2_(H_2_O)_6_, 0.5 mM (NH_4_)_2_CO_3_, 0.075 mM CaCl_2_. The pH was adjusted to 3.0 with 6 M HCl. Subsequently, 2,000 U/ml of pepsin in 2 ml of SGF was added to initiate the digestion process. This process was carried out at 37°C for 2 h in a water bath with constant shaking at 150 rpm. After 2 h of digestion, potassium carbonate (1.0 M) was added to adjust the mixture to pH 7.0 to deactivate pepsin. The mixture was then transferred into a Snakeskin dialysis tubing with a molecular weight cut-off at 7 kDa.

Following the gastric phase digestion, small intestinal phase digestion was stimulated. Simulated intestinal fluid (SIF) was prepared freshly before use according to (12) with the following concentration of salts: 6.8 mM KCl, 0.8 mM KH_2_PO_4_, 85 mM NaHCO_3_, 38.4 mM NaCL, 0.33 mM MgCl_2_(H_2_O)_6_, 0.3 mM CaCl_2_(H_2_O). The pH was adjusted to 7.0 with 1M NaOH. The small intestinal digestion was initiated by adding 100 U/mL of pancreatin in 4 ml of SIF and 10 mM of bile into the mixture. After which, the dialysis tube was placed in a 100 ml sodium phosphate buffer (0.1 M, pH 7.0) fortified with calcium chloride (40 mg/L). This digestion was carried out in a shaker water bath (37°C) under continuous shaking at 150 rpm for 3 h. During the 3-h duration, aliquots (200 μl) of dialysate were withdrawn at different time intervals (0, 10, 20, 30, 40, 50, 60, 90, 120, 150 and 180 min) and immediately replaced with a fresh sodium phosphate buffer (200 μl). For all sets of experiments, a blank sample was prepared using denatured enzymes.

### Determination of Inhibition Activity Using DNSA

DNSA was utilised to measure the amount of reducing sugar that is released from the dialysate (13). DNSA reagent (100 μl) and aliquots of dialysate (100 μl) were mixed in each well of a 96-well microplate and steamed at 100°C for 10 min until a reddish-brown colour was developed. The microplate was subsequently cooled to room temperature in an ice-water bath. Thereafter, 100 μl of mixture solution was transferred to a new 96-well microplate reader and the absorbance was read at 540 nm using a microplate absorbance reader (Biotek Instruments Inc., Winooski, VT, USA). Maltose equivalent (ME) was calculated from a pre-determined maltose calibration curve of *y* = 0.6022 *x*-0.1738 where *y* is the absorbance at 540 nm and *x* is the concentration of maltose in mg/ml. The degree of hydrolysis was then expressed as milligram ME per gram of starch. The percentage inhibition of flour digestion over 3 h was calculated using Equation 3. AUC_control_ and AUC_sample_ represent the area under the curve obtained from the maltose released vs. time graph.

(3) $$\begin{array}{c} \text{Percentage Inhibition}=\left({\text{AUC}}_{\text{control}}-\frac{{\text{AUC}}_{\text{sample}}}{{\text{AUC}}_{\text{control}}}\right)\times 100\% \end{array}$$

### Determination of Starch Content

Starch content of the wheat and rice flour was determined using the AOAC method 991.43 (14). Each sample was prepared by adding 1 g of flour samples into 25 ml of sodium phosphate buffer (pH 6.0, 0.08 M). After which, 50 μl of α-amylase (thermo-stable, 3,000 U/ml) was added to all samples and subsequently incubated in an 80°C water bath for 25 min with constant shaking at 150 rpm. Thereafter, the pH value of all samples was adjusted to pH 7.5 before protease (0.1 ml) was added. The samples were then incubated at 60°C for 40 min. After 40 min of incubation, the mixture was adjusted to pH 4.5 and 0.2 ml of amyloglucosidase was added. After the second round of incubation at 60°C was carried out for 40 min, ethanol (95%, 140 ml) was added and the mixtures were left overnight. Celite was wet with ethanol (78%) and even out on a crucible. The samples were then filtered and kept for starch analysis using DNSA assay at 540 nm as described in setion Characterisation of compounds by high performance liquid chromatography-mass spectrometry. The absorbance was used to calculate the starch content using a predetermined glucose calibration curve, y = 1.7702x – 0.1731 with a R^2^ value of 0.999, where y represents the absorbance at 540 nm and x represents the concentration of glucose solution in g/L.

### Colour Analysis of Cooked Noodles

The colour of the cooked rice and wheat noodles were measured using a spectrophotometer (Konica Tokyo, Japan) that was equipped with D65 illuminant based on the CIE 1976 (L^*^, a^*^, b^*^) colour space. The measurement area was set to 8 mm and the spectrophotometer was set to exclude the specular component for all measurements.

### Textural Analysis of Cooked Noodles

The textural analysis of the cooked noodles was tested using a texture analyser (TA-XT2i; Stable Micro System Ltd., Godalming, Surrey, UK) (11). The hardness and adhesiveness of the cooked noodle strands with 2 mm thickness and 1 cm width were determined through compressing the noodles using a cylinder probe with a diameter of 35 mm. The TA-XT2i settings were as follows: pre-test speed (1.0 mm/s), test speed (1.0 mm/s), post-test speed (3.0 mm/s), strain (75 %), time (2 s), trigger force (5 g). The chewiness of the noodle was determined from the force-time curve of the texture profile. On the other hand, extensibility and tensile strength of 15 cm noodles with 2 mm thickness and 1 cm width were tested by using an A/SPR probe (Spaghetti/noodle tensile rig). The settings of the analyser were as follows: pre-test speed (1.0 mm/s), test speed (3.0 mm/s), post-test speed (10.0 mm/s), distance (100 mm), trigger force (5 g). The distance between the two arms of the rig was set at 40 mm. The measurements were repeated three times for each sample.

### Statistical Analysis

All experiments were carried out in triplicates. The results are given as mean ± standard deviations. One-way analysis of variance (ANOVA) with TUKEY's test (*p* < 0.05) was used to determine the differences within the groups using SPSS software.

## Results and Discussion

### Inhibition Activities of EA Extract of *P. viridiflora* Stems Against α-Amylase or α-Glucosidase

**Figure 1:**
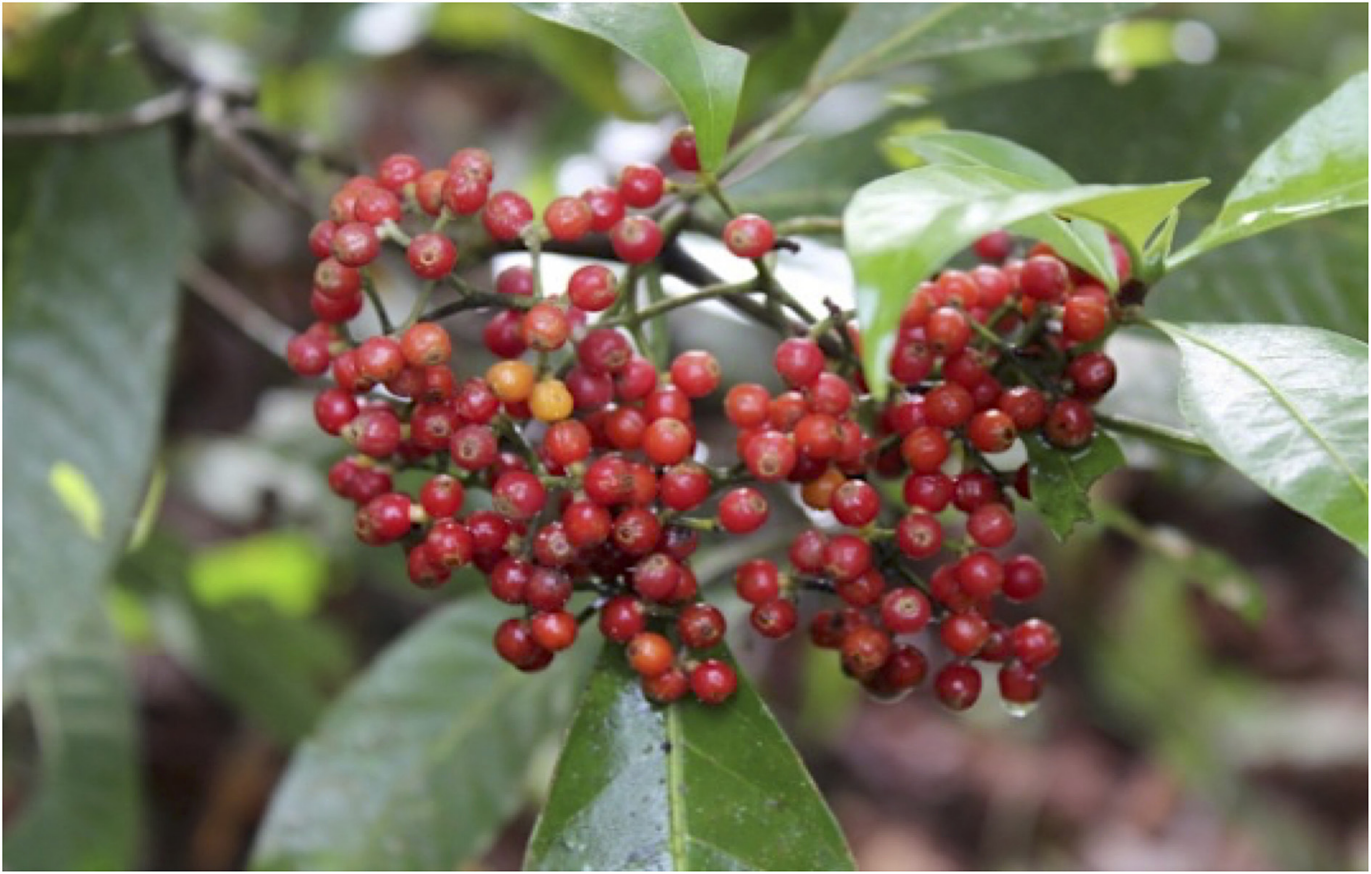
An image of *P. viridiflora*.

*P. viridiflora* is locally known as Engkerabai by the Ibans, an indigenous community from Rumah Bajau, Julau, Sarikei Division. The taxonomy identification (Voucher specimen: SABC 10761) was carried out based on the plant characteristics recorded in the specimens at Sarawak Herbarium (SAR) from the Forest Department Sarawak and the appearance of the *P. viridiflora* is shown in Figure 1. Currently, no scientific research has been carried out on this native plant from Sarawak.

**Figure 2:**
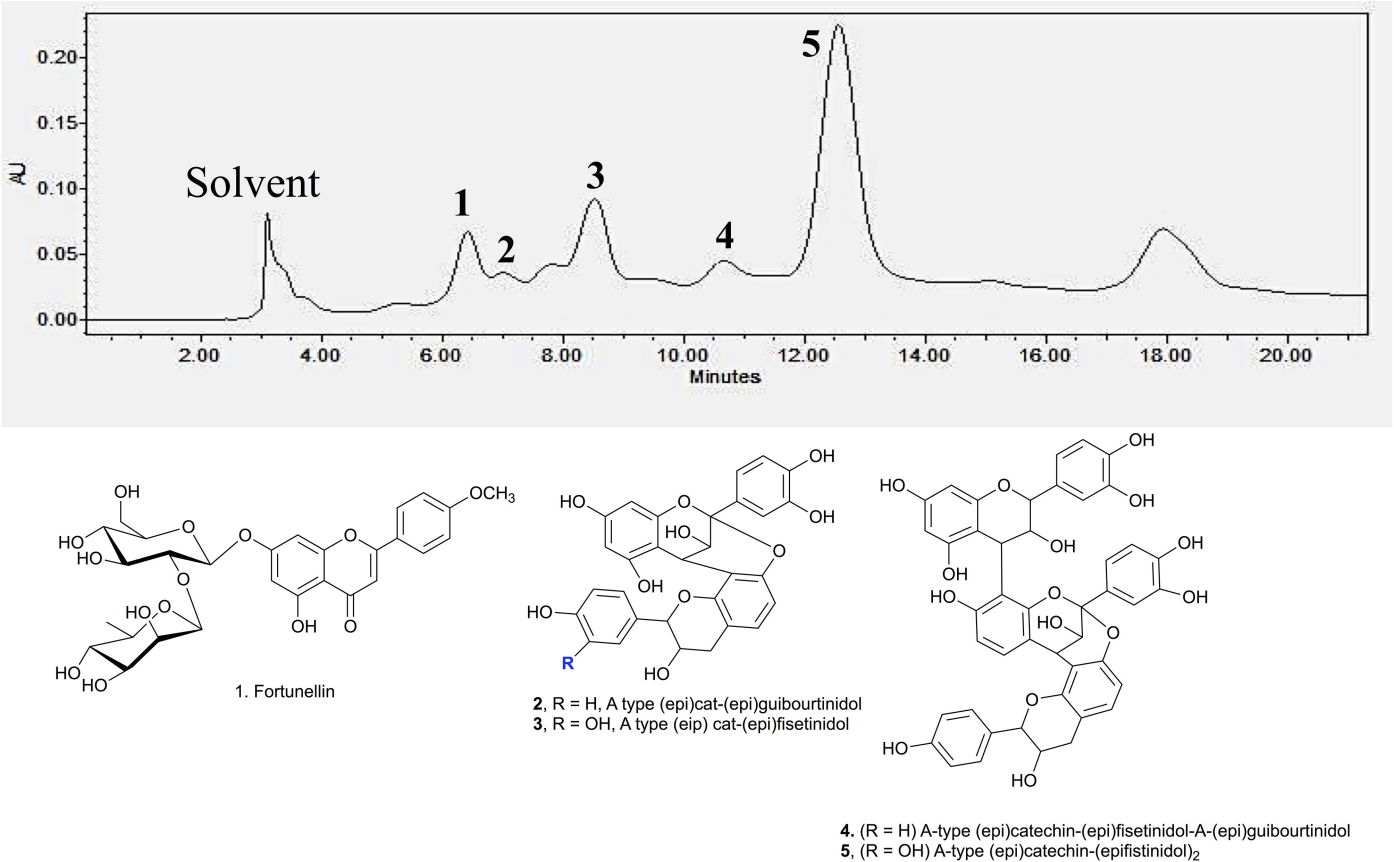
Fragmentation pattern of MS(n) for peak 3 at 8.5 min.

A total of six different solvents (hexane, methanol, acetone, water, ethyl acetate, dichloromethane) were used for the extraction process and the inhibition activities of the extracts were evaluated. Since the IC_50_ values are dependent on many factors such as enzyme origin, the concentration of substrates and other factors like pH, temperature and the buffer being used, it is more accurate to compare the AE values instead. In fact, a higher AE indicates higher inhibitory activity. In general, only acetone and ethyl acetate fractions exhibited inhibition activities while no inhibition effects were observed for hexane, methanol, water and dichloromethane extracts against α-amylase and α-glucosidase. The AE values of the acetone extract against α-amylase and α-glucosidase were 0.115 (IC_50_ = 40.0 μg/ml) and 0.020 (IC_50_ = 115.0 μg/ml), respectively. On the other hand, the AE of the EA extract against α-amylase and α-glucosidase were 0.288 ± 0.128 (IC_50_ = 15.4 ± 2.1 μg/ml) and 0.095 ± 0.031 (IC_50_ = 32.4 ± 3.7 μg/ml), respectively (Figure 2). These results illustrate that the extract obtained from EA exhibits higher inhibition effects as compared to the acetone extract.

To date, various α-amylase and α-glucosidase inhibitors from natural sources like plants have been identified and characterised (15–17). For instance, grape seed extract, containing procyanidins was reported to possess an AE value of 0.793 (IC_50_ = 8.7 ± 0.8 μg/ml) against α-amylase and an AE value of 75.8 (IC_50_ = 1.2 ± 0.2 μg/ml) against α-glucosidase (18) which were much higher than *P. viridiflora* extract. In contrast, the AE values of green tea and white tea extracts (rich in tea catechins) against α-amylase were 0.197 (IC_50_ = 34.9 ± 0.9 μg/ml) and 0.018 (IC_50_ = 378.0 ± 134.0 μg/ml), respectively (18) and were much lower than EA extract of *P. viridiflora*. On the other hand, the inhibition effect of these tea extracts against α-glucosidase were remarkably higher as compared to *P. viridiflora*. In general, the AE value of the green tea extract against α-glucosidase was 182.0 (IC_50_ = 0.5 ± 0.1 μg/ml) and the AE value of the white tea extract against α-glucosidase was 36.4 (IC_50_ = 2.5 ± 0.4 μg/ml) which were notably higher than the EA extract of *P. viridiflora*. Apart from procyanidins, fucoidan that is found in brown marine algae (*Ascophyllum nodosum*) was also reported to have a α-glucosidase inhibition effect with an AE value which ranges from 21.2 to 76.9 (IC_50_ = 13.0 to 47.0 mg/ml) (19). However, no α-amylase inhibition effects were detected from fucoidan obtained from certain algae species like *Fucus vesiculosus* (19). Taken together, these studies reveal that the α-amylase inhibition effect of *P. viridiflora* extract is comparable or even higher than some commonly known plant extracts. On the other hand, the α-glucosidase inhibition effect may be slightly weaker as opposed to certain plant sources. The difference might be due to the concentration of inhibitors in the extracts.

### Characterisation of Bioactive Compounds by HPLC-MS

**Figure 3:**
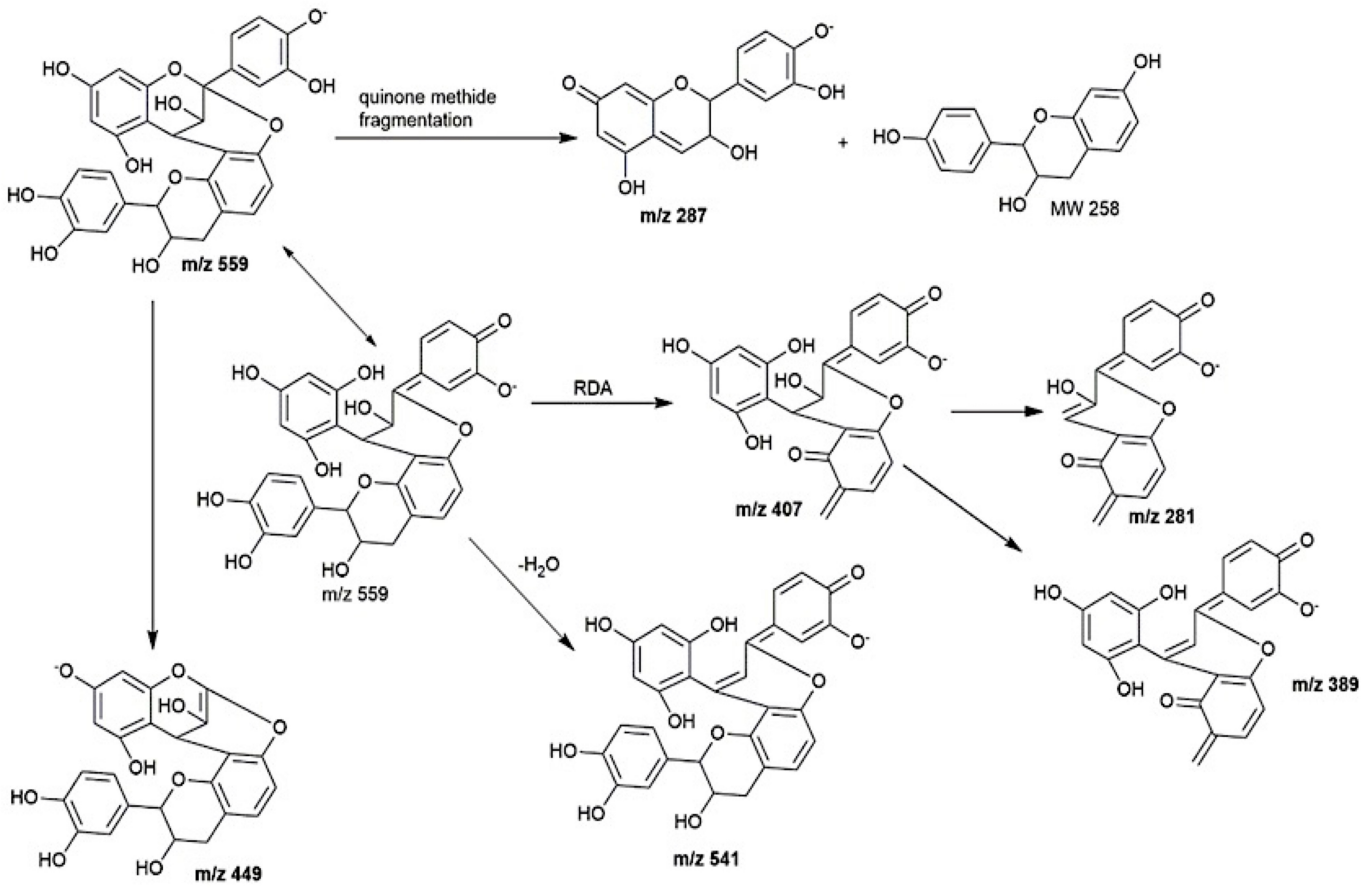
HPLC chromatogram of EA fraction under UV 280 nm and the compounds associated to the major peaks.

Identification of the active compounds responsible for the starch hydrolase inhibition activity were performed through HPLC-ESI-MS/MS. Additionally, Develosil diol column was utilised for the HPLC analysis due to its good baseline resolution to separate bioactive compounds like proanthocyanidins. Figure 3 shows the HPLC chromatogram of the EA fraction with the tentatively assigned proanthocyanidins based on the HPLC-ESI-MS/MS results (the MS data for each peak are shown in Supporting Information). Conclusive assignments of their structures would require further separation of each compound and characterisation by single crystal X-ray diffraction.

Peak **1** at 6.7 min was found to be fortunellin with *m/z* value of 593.2 [M-H]^−^ and MS^2^ ions at *m/z* 511, 409, and 289 which is consistent with literature reported fragmentation patterns. To date, limited research has been conducted on Fortunellin. This compound exhibits both anti-inflammation and anti-oxidative activity in a diabetic mice model by regulating the AMPK/Nrf-2 Pathway (20). Fortunellin can also maintain the intestinal barrier function and decrease inflammation in the colitis (21). In view of these properties, fortunellin may be partially responsible for the health promotion activity of *P. viridiflora* stem.

Peaks **2** to **6** contain dimeric or trimeric proanthocyanidins, which are composed of different combinations of (epi)catechin, (epi)fisetinidol, (epi)afzelechin, and (epi)guibourtinidol).While procyanidins containing (epi)catechin is most widely found in the plant kingdom, propelargonidins containing (epi)afzelechin is comparatively rare (22). Likewise, only a few plant species containing (epi)guibourtinidol and (epi)fisetinidol monomers that are found in proguibourtinidins and profisetinidin, respectively, have been discovered (22, 23). The peaks were tentatively assigned to contain different combinations of identified monomers based on their MS and MS^n^ values in conjunction with those reported in the literature (24, 25). From the LC-MS results, dimeric compounds can be identified through the characteristic fragmentation patterns of proanthocyanidins due to quinone methide (QM) reaction, heterolytic ring fission (HRF) and retro-diels-alder reaction (RDA) (26).

**Figure 4:**
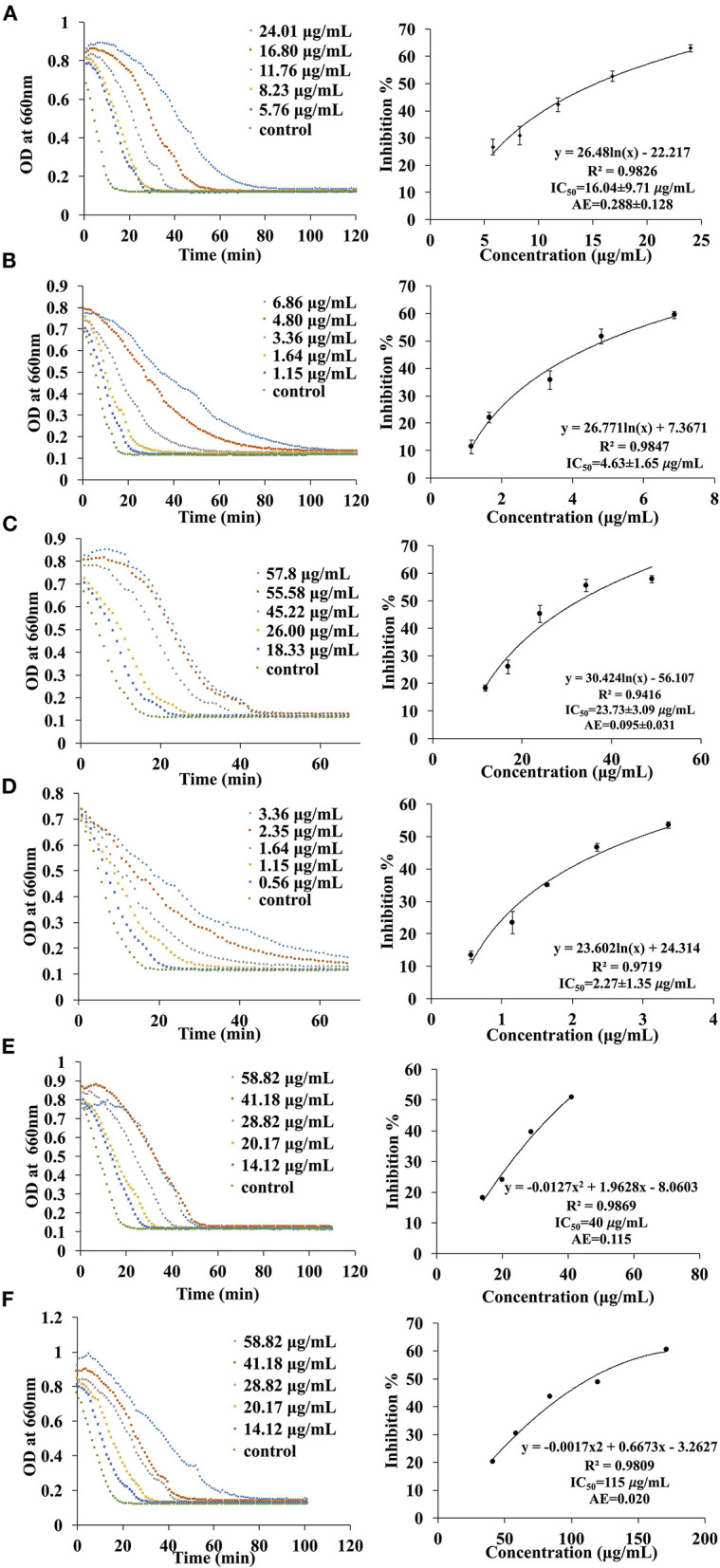
The kinetic curve and dose responsive curve of starch hydrolase in the presence of different concentrations of **(A)** EA extract of *P. viridiflora* against pancreatic α-amylase, **(B)** acarbose against pancreatic α-amylase, **(C)** EA extract of *P. viridiflora* against α-glucosidase, **(D)** acarbose against α-glucosidase, **(E)** acetone extract of *P. viridiflora* against pancreatic α-amylase, **(F)** acetone extract of *P. viridiflora* against alpha-glucosidase.

Peak 2 at 7.4 min gave *m/z* value of 543 [M-H]^−^ and MS^2^ ions at *m/z* 525, 433, 407. Daughter ion *m/z* 407 [M-H-136]^−^ was fragmented due to RDA fragmentation. Loss of 136 Da implies that there was one hydroxyl group at ring B of the extension unit. Additionally, daughter ions at *m/z* 433 [M-H-126]^−^ were obtained due to HRF fragmentation. The loss of 126 Da indicates a 1,3,5-trihydroxybenzene structure at ring A of the extension unit (26). Therefore, HRF pathway indicates the existence of an OH group on ring B which is deduced to be (epi)afzelechin. Taken together, we propose peak 2 to be an A-type (epi)fisetinidol-(epi)afzelechin. Peak **3** at 8.5 min gave *m/z* value of 559 [M-H]^−^ and MS^n^ ions at *m/z* 541, 449, 407, 389, 287. Based on the MS^n^ fragmentation pattern analysis (shown in Figure 4), we assigned the identity of the compound to be an A-type dimer of (epi)catechin-fisetinidol dimer (27).

Peak **4** at 11.4 min showed a group of molecular ions at *m/z* 799, 815, 831, and 877. They can be assigned to be trimeric proanthocyanidins with (epi)catechin-(epi)fzalechin-A-(epi)guibourtinidol (molecular weight of 800), and its homologues with one or more OH groups. Taking the MS (2) of the peak at 815 as an example, it fragmented into *m/z* 705, 663, 575, 559, 527, 511, 407, 389, and 287. These fragmentation patterns are observed in typical oligomeric proanthocyanidins MS fragmentations. Fragment 287 is due to quinone methide cleavage of the (epi)catechin terminal unit. For the peak at *m/z* 705 [M-H-110]^−^, HRF fragmentation (–C_6_H_6_O_2_) indicates the presence of an extension unit with a 1,3-dihydrobenzene structure at ring A. The fragment at *m/z* 663.1 [M-H-152]^−^ is due to RDA fragmentation. Loss of 152 Da through RDA indicates that the ring B of the extension unit has two hydroxyl groups which correspond to an (epi)fisetinidol unit. Fragment ion at *m/z* 527 resulted from losing a neutral piece that contains a molecular weight of 288 from the mother ion. Peak **5** at 12.2 min is a trimer with a molecular anion at 831 which indicates the presence of an A-type (epi)catechin-((epi)fisetinidol)_2_.

Therefore, based on the LC-MS results of the EA extracts, we can conclude that this plant contains mostly A-type dimeric and trimeric proanthocyanidins, which are known starch hydrolase inhibitors.

#### *In vitro* Digestion of Rice and Wheat Noodles

**Figure 5:**
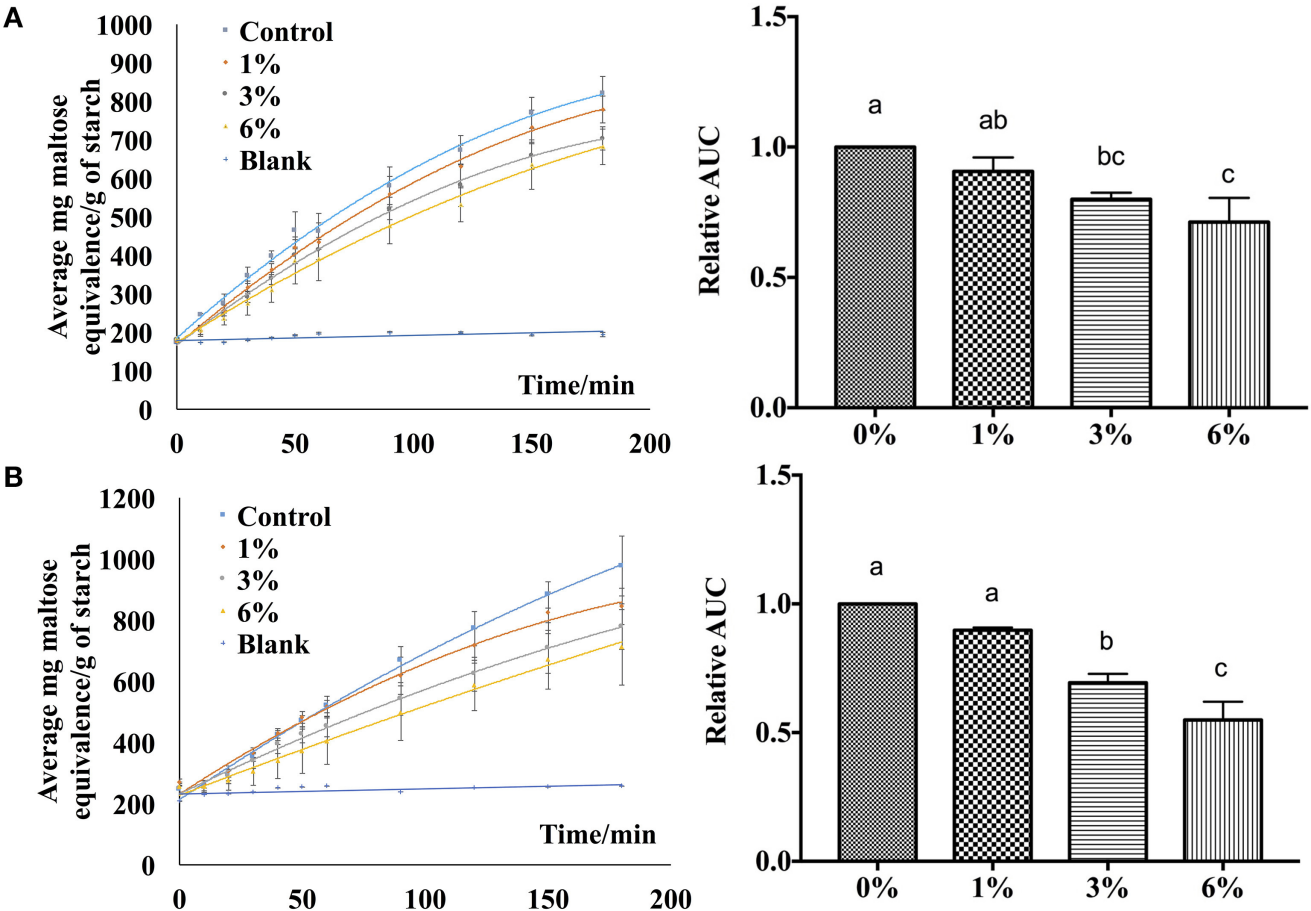
Digestibility profile of **(A)** wheat noodles and **(B)** rice noodles. Figures on the left are expressed as mg maltose equivalence/g of starch in flour during *in vitro* digestibility analysis for 180 min while figures on the right are expressed using the area under the curve. ^a, b, c^ANOVA (*n* = 3) at 95% confidence level with same letters indicating no significant difference in area under the curve (AUC) at different concentrations.

In order to verify the effectiveness of the starch hydrolase inhibition activity of *P. viridiflora* plant extract, EA extract was incorporated into rice and wheat noodles and a simulated digestion process was performed using an *in vitro* digestion model. As shown in Figure 5, when a higher concentration of EA extract was incorporated into both rice and wheat noodles, a slower release of maltose was observed. Additionally, control samples have a higher starch hydrolysis rate as compared to noodle samples which are incorporated with the EA extracts. This indicates that a higher starch hydrolase inhibition activity was detected when a higher concentration of EA extract was incorporated.

Additionally, the area under the curve was calculated to determine the digestibility of noodles after incorporating EA extract at different concentrations (Figure 5). An overall decrease in AUC was observed as the extract concentration increases for both rice and wheat noodles which suggests a dose-dependent relationship on the digestibility of the two noodles since an increase in EA extract from 1 to 6% results in a decline in digestibility.

Based on the calculated AUC, the inhibition activity of wheat and rice noodles digestion was determined (Table 1). The digestibility of wheat noodles containing 1, 3, and 6% of the EA extract were reduced by 8.77 ± 3.64%, 19.77 ± 1.69%, and 27.25 ± 6.77%, respectively. On the other hand, the digestibility of the rice noodles when incorporated with 1, 3, and 6% of EA extract showed a reduction by 9.14 ± 1.64%, 26.89 ± 5.95%, and 37.16 ± 15.29%, respectively. In general, rice noodles had a higher inhibition activity as compared to wheat noodles after the addition of EA extract. This could be caused by the differences in protein content between the two different types of flour which could interact with proanthocyanidins during the noodle making and cooking process. Since wheat flour contains a higher protein content (10.5%) as compared to rice flour (6.5%), the presence of proteins will interfere with the digestibility of the noodles by binding to proanthocyanidins and thereby reducing its bioavailability. As such, this results in wheat noodles having a lower starch hydrolase inhibition activity as compared to rice noodles despite possessing the same percentage of EA extract.

**Table 1:** Percentage inhibition of wheat and rice noodles digestion.

**EA fraction**	**% Inhibition**	
	**Wheat**	**Rice**
1%	8.77^a^	9.14^a^
3%	19.77^ab^	26.89^ab^
6%	27.25^b^	37.16^b^

a,b *ANOVA at 95% confidence level with same letters indicating no significant difference in the percentage inhibition at different concentration of EA fraction*.

### Moisture Content Analysis

The moisture content of the rice and wheat noodles were measured. As shown in Table 2, with an increase in EA extract concentration from 1 to 6% (w/w), the moisture content for both wheat and rice noodles increased slightly. Additionally, no significant differences (*p* > 0.05) in moisture content were observed in the presence of 1 and 3% of the extract in wheat noodles. On the other hand, wheat noodles with 6% EA extract showed the highest moisture content (56.4 ± 0.3%) while the control exhibits the lowest moisture content (52.8 ± 0.2%). Likewise, rice noodles containing the EA extract exhibit a similar trend whereby 6% extract showed the highest moisture content (64.2 ± 0.5%) which is significantly different from the rest (*p* < 0.05). This observation may be due to a decrease in total flour composition in the noodles as the percentage of EA extract increases. Henceforth, less flour is available in the noodle matrix to absorb water in the presence of a higher EA extract concentration. Overall, the moisture content of the control rice noodles (60.6 ± 0.5%) was generally higher than the control wheat noodles (56.4 ± 0.3%). This difference in moisture content is probably due to the starch and protein content differences within the two flours.

**Table 2:** Moisture content of noodles incorporated with EA extract.

**Noodles**	**EA extracts**			
	**0%**	**1%**	**3%**	**6%**
Wheat	52.8 ± 0.2^a^	54.1 ± 0.7^b^	54.4 ± 0.3^b^	56.4 ± 0.3^c^
Rice	60.6 ± 0.5^a^	61.3 ± 0.3^a^	62.8 ± 0.6^b^	64.2 ± 0.5^c^

a,b,c *Means within rows followed by different letters are significantly different (p < 0.05)*.

### Colour Analysis

**Figure 6:**
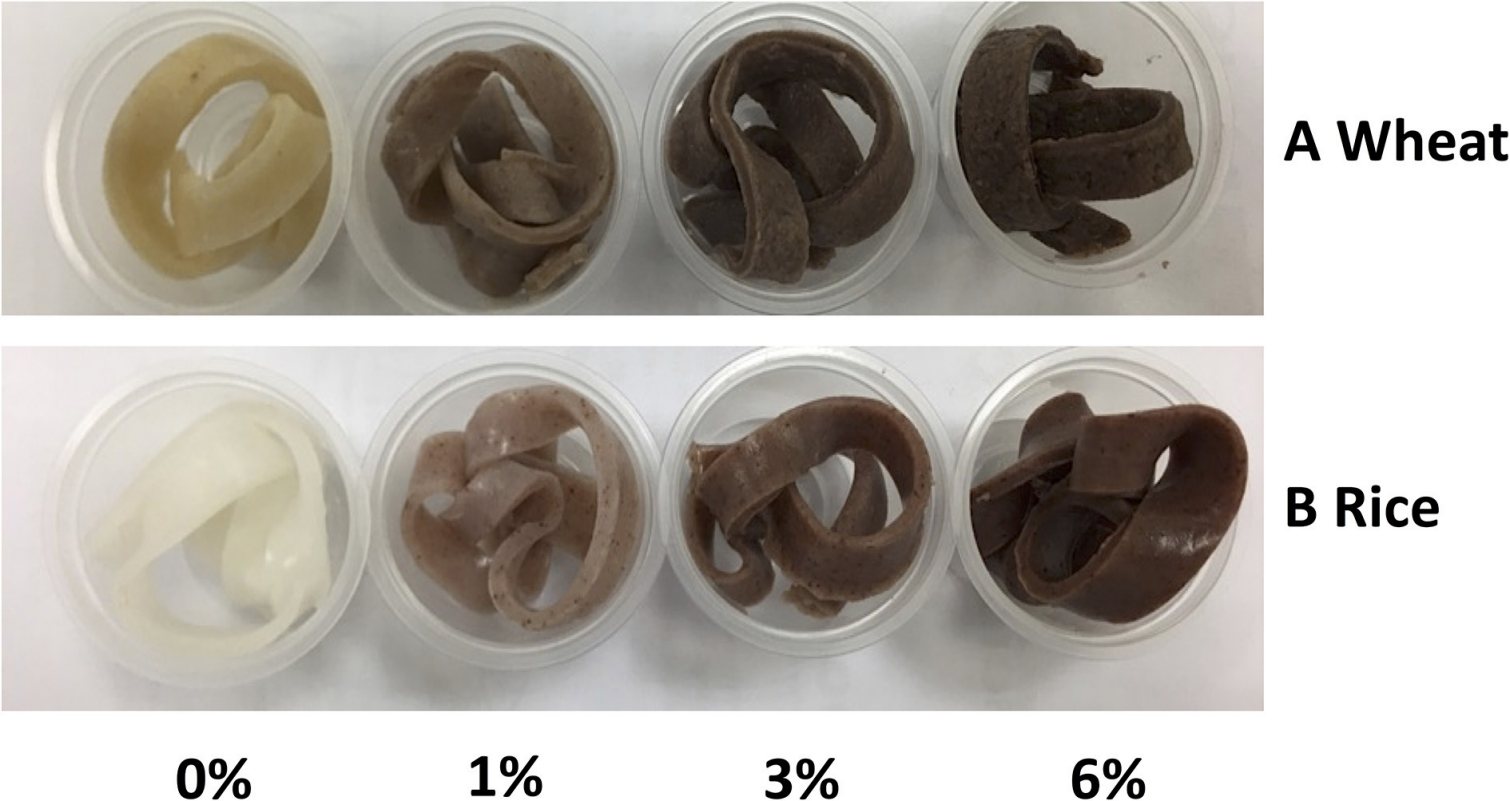
Colour swatches for cooked **(A)** Wheat and **(B)** Rice noodles incorporated with EA extract of different dosages (0%, 1%, 3% and 6%).

Colour is one of the major determinants of noodle marketability. In Table 3, three parameters were tested whereby L^*^ stands for lightness, a^*^ stands for redness and greenness while b^*^ stands for yellowness and blueness. In general, with an increased in EA extract from 0 to 6% (w/w), the cooked noodles showed significant changes in the L^*^, a^*^, and b^*^ values (*p* < 0.05). For instance, the lightness of the noodles decreased with an increased in EA extract. Since the EA extract is dark brown, the addition of these extracts will lead to an increased in darkness and a decreased in brightness of the noodles (Figure 6). Due to the colour of the extract, a^*^ values which represent redness and greenness also increased significantly for both noodles. As for b^*^ value (yellowness and blueness), wheat noodles showed a significant decrease while rice noodles showed a slight increase with increased extract concentration. This is because wheat flour is slightly yellow and with the addition of the reddish-brown extract, the darker shade of brown will overpower the light yellow colour in the wheat flour (28).

**Table 3:** Colour properties of cooked rice and wheat noodles incorporated with EA extract.

**Noodles**	**Colour**		
	L*(D65)	a*(D65)	b*(D65)
W0%	62.77 ± 0.04^a^	2.44 ± 0.01^a^	19.33 ± 0.02^a^
W1%	44.99 ± 0.03^b^	6.89 ± 0.02^a^	10.31 ± 0.02^b^
W3%	37.88 ± 0.02^c^	8.16 ± 0.01^b^	9.44 ± 0.02^c^
W6%	34.71 ± 0.02^d^	8.46 ± 0.03^c^	10.49 ± 0.02^d^
R0%	69.55 ± 0.02^a^	−0.59 ± 0.01^a^	7.14 ± 0.04^a^
R1%	46.01 ± 0.01^b^	8.90 ± 0.01^b^	9.01 ± 0.01^b^
R3%	35.97 ± 0.01^c^	10.60 ± 0.02^c^	9.53 ± 0.02^c^
R6%	32.61 ± 0.01^d^	10.20 ± 0.02^d^	9.40 ± 0.02^d^

a,b,c,d *ANOVA (n = 3) at 95% confidence level with same letters indicating no significant difference*.

*W = wheat noodle; R = rice noodle*.

L*, a*, and b* are hunter L, a, and b values.

Generally, functional food products like noodles have become more colourful in recent years. In fact, food that are darker in colour like brown rice are perceived as nutritious to consumers. Research has also shown that red and yellow coloured food could subconsciously help to attract attention from consumers while blue and green coloured food is often associated with food that is healthy (29). Since noodles incorporated with EA extracts of *P. viridiflora* show an increase in a^*^, these noodles could potentially help in stimulating consumers' appetite (29).

### Textural Profile Analysis

Textural attributes of cooked noodles were evaluated by texture profile analysis. Attributes including hardness, chewiness, adhesiveness, tensile strength, and extensibility were analysed. On the whole, the incorporation of EA extracts into the noodles will show several effects on the textural properties. This measurement is imperative to gauge the changes in organoleptic properties which will tend to affect the consumer acceptance of cooked noodles. From Table 4, no significant differences were observed for both hardness and adhesiveness of the wheat and rice noodles despite an increased in extract concentration (*p* > 0.05). However, in comparison to wheat noodles, rice noodles were reported to have a lower adhesiveness and a higher extensibility value.

**Table 4:** Textural properties of cooked noodles incorporated with EA extracts.

**Noodles**	**Textural properties**				
	**Hardness(g)**	**Chewiness (g)**	**Adhesiveness (g s)**	**Tensile strength (g)**	**Extensibility (mm)**
W0%	27222.05 ± 5603.02^a^	11938.41 ± 1727.37^a^	−1323.58 ± 261.47^a^	159.59 ± 8.22^a^	69.78 ± 15.80^a^
W1%	21116.9 ± 1965.33^a^	8060.95 ± 8060.94^ab^	−1141.22 ± 2.26^a^	123.98 ± 0.30^b^	56.30 ± 3.94^a^
W3%	23289.23 ± 1784.37^a^	7305.06 ± 1116.65^b^	−1261.55 ± 14.87^a^	82.35 ± 12.05^c^	29.15 ± 9.34^b^
W6%	25855.26 ± 1095.92^a^	7248.78 ± 1553.79^b^	−1066.9 ± 64.37^a^	77.75 ± 2.49^c^	18.70 ± 2.20^b^
R0%	24743.94 ± 2527.74^a^	17568.87 ± 2844.08^a^	−160.11 ± 118.48^a^	177.84 ± 24.81^a^	26.98 ± 1.18^a^
R1%	25104.41 ± 2161.74^a^	17901.22 ± 133.84^a^	−192.77 ± 99.08^a^	95.41 ± 1.66^b^	24.85 ± 1.03^ab^
R3%	29532.83 ± 5125.49^a^	19094.58 ± 2918.63^a^	−249.7 ± 82.99^a^	86.78 ± 16.87^b^	18.90 ± 5.66^bc^
R6%	30800.06 ± 2624.91^a^	20921.17 ± 2462.71^a^	−236.51 ± 75.35^a^	70.91 ± 14.51^b^	15.04 ± 3.29^c^

a,b,c *ANOVA at 95% confidence level with different letters indicating significant difference (p < 0.05). Data expressed as mean± SD of triplicate determination. W = wheat noodle;R = rice noodle*.

In general, the chewiness, tensile strength, and extensibility values of EA extract incorporated into wheat noodles have all decreased. The decreased in chewiness, tensile strength, and extensibility values with an increased in EA extract concentration indicates that the wheat noodles will become less stretchable and will break easily under force. On the other hand, chewiness value increased, while tensile strength and extensibility values decreased when EA extract concentration in rice noodles increased. From Table 4, significant differences in chewiness value were observed between higher concentrations of the EA extract (3 and 6%) and the control wheat noodles. Although a slight increase in chewiness could be observed in the rice noodles with an increasing concentration of extract, this difference is not significantly different (*p* < 0.05). Additionally, the tensile strength of both rice and wheat noodles also decreases significantly with increased concentration of EA extract. However, no significant differences in the tensile strength were observed between wheat noodles containing 3 or 6% of the EA extract. Likewise, no significant differences were observed between the tensile strength of rice noodles with 1, 3%, or 6% of the EA extract. Extensibility values of both the wheat and rice noodles decrease with increasing EA extract concentrations. This is because gluten network formation will be negatively impacted at high EA extract concentrations, thus decreasing the extensibility of noodles. A significant difference in the extensibility value could be observed between the control noodles and noodles with high EA extract concentrations (3 and 6%).

Overall, the incorporation of the EA extract had imposed some significant differences on the textural properties of different types of cooked noodles. For instance, changes in textural properties were observed as the wheat noodles became softer, less chewy and less adhesive with an increased level of EA extract. Rice noodles, on the other hand, were more firm, adhesive and chewier with increasing concentration of EA extracts.

## Conclusions

In conclusion, our data suggest that the EA extracts of *P. viridiflora* could be a viable alternative to inhibit α-amylase and α-glucosidase. In comparison to other plant extracts (green tea, white tea), the α-amylase inhibition activity of *P. viridiflora* is higher while the α-glucosidase inhibition activity is lower (18). Although proanthocyanidins with a low degree of polymerisation were identified to be the major compounds within the extract, future work is required to determine if specific proanthocyanidins are responsible for the inhibitory effects. Overall, the quality attributes of the noodles as well as the digestibility of the noodles were influenced by the addition of *P. viridiflora* extract. In general, the fortification of *P. viridiflora* in the formulation of functional noodles will help to provide a healthier alternative to diabetic patients.

## Data Availability Statement

The original contributions presented in the study are included in the article/Supplementary Material, further inquiries can be directed to the corresponding author/s.

## Author Contributions

The planning of the research was done by QC, DH, CS, and TY. The experiments were performed by JT and QC. All the writers have contributed in the research work and the write-up of this article.

## Conflict of Interest

The authors declare that the research was conducted in the absence of any commercial or financial relationships that could be construed as a potential conflict of interest.

## Publisher's Note

All claims expressed in this article are solely those of the authors and do not necessarily represent those of their affiliated organizations, or those of the publisher, the editors and the reviewers. Any product that may be evaluated in this article, or claim that may be made by its manufacturer, is not guaranteed or endorsed by the publisher.
