# Supplementary material for: Inhibition Effect of Extract of Psychotria viridiflora Stem on α-Amylase and α-Glucosidase and Its Application in Lowering the Digestibility of Noodles
Source: Front Nutr. 2021 Aug 12;8:701114. doi: 10.3389/fnut.2021.701114 (PMC8387799; doi:10.3389/fnut.2021.701114)
Supplement: Supplementary file 1 [file Data_Sheet_1.DOCX]

## Inhibition effect of extract of *Psychotria viridiflora* stem on α-amylase and α-glucosidase and its application in lowering the digestibility of noodles

## Qimin Chen^1a^, Joanne Yi Hui Toy^1a^, Cynthia Seta^3^, Yeo Tiong Chia^3^, Dejian Huang ^1,2,*^

## ^1^ Department of Food Science and Technology, National University of Singapore, Science Drive 2, Singapore 117542, Singapore.

## ^2^ National University of Singapore (Suzhou) Research Institute, 377 Lin Quan Street, Suzhou Industrial Park, Jiangsu 215123, People’s Republic of China

^3^Sarawak Biodiversity Centre, KM20, Jalan Borneo Heights, Semengoh, Locked Bag No. 3032, 93990, Kuching, Sarawak, Malaysia

a, these authors contributed equally, *Corresponding author: Dejian Huang E-mail address: dejian@nus.edu.sg

**SUPPORTING INFORMATION**

**Figure Captions:**

**Figure S1:** LC-MS spectral data for peak 2 shown in Figure 2 at 7.3 min.

**Figure S2:** LC-MS spectral data for peak 3 shown in Figure 2 at 8.7 min.

**Figure S3:** LC-MS spectral data for peak 4 shown in Figure 2 at 11.4 min.

**Figure S4:** LC-MS spectral data for peak 5 shown in Figure 2 at 12.2 min.

**Figure S5:** HR-LCMS spectrum (negative mode) for ethyl acetate extract of *Psychotria Viridiflora* stem for peak at 834, the main flavone trimer (peak 5).

**Figure S1:** LC-MS spectral data for peak 2 shown in Figure 2 at 7.3 min.




**Figure S2:** LC-MS spectral data for peak 3 shown in Figure 2 at 8.7 min.

**Figure S3:** LC-MS spectral data for peak 4 shown in Figure 2 at 11.4 min.

**Figure S4:** LC-MS spectral data for peak 5 shown in Figure 2 at 12.2 min.

 **Figure S5:** HR-LCMS spectrum (negative mode) for ethyl acetate extract of *Psychotria Viridiflora* stem for peak at 834, the main flavone trimer (peak 5).
